# Stochastic Motion Stimuli Influence Perceptual Choices in Human Participants

**DOI:** 10.3389/fnins.2021.749728

**Published:** 2022-03-02

**Authors:** Pouyan R. Fard, Sebastian Bitzer, Sebastian Pannasch, Stefan J. Kiebel

**Affiliations:** ^1^Department of Psychology, Technische Universität Dresden, Dresden, Germany; ^2^Centre for Tactile Internet With Human-in-the-Loop (CeTI), Technische Universität Dresden, Dresden, Germany

**Keywords:** perceptual decision making, random-dot motion task, Bayesian inference, drift-diffusion model, model comparison

## Abstract

In the study of perceptual decision making, it has been widely assumed that random fluctuations of motion stimuli are irrelevant for a participant’s choice. Recently, evidence was presented that these random fluctuations have a measurable effect on the relationship between neuronal and behavioral variability, the so-called choice probability. Here, we test, in a behavioral experiment, whether stochastic motion stimuli influence the choices of human participants. Our results show that for specific stochastic motion stimuli, participants indeed make biased choices, where the bias is consistent over participants. Using a computational model, we show that this consistent choice bias is caused by subtle motion information contained in the motion noise. We discuss the implications of this finding for future studies of perceptual decision making. Specifically, we suggest that future experiments should be complemented with a stimulus-informed modeling approach to control for the effects of apparent decision evidence in random stimuli.

## Introduction

A key question in perceptual decision making is how the brain rapidly makes decisions to categorize sensory input. Many different tasks have been used to investigate perceptual decision making but one that stands out due to its wide-spread use in human and animal experiments is the so-called random-dot motion (RDM) task (Newsome and Pare, 1988; Britten et al., 1996). The RDM task is a motion direction discrimination task, usually applied in two-alternative forced choice settings, where participants must decide about the net motion direction of a cloud of seemingly randomly moving dots presented on the screen. The RDM task was instrumental in unraveling many aspects of the underlying neural and behavioral mechanisms of perceptual decision making, both in humans and animals (see Gold and Shadlen, 2007 for review).

RDM stimuli are usually generated by embedding coherently moving dots in a backdrop of randomly appearing dots. The fraction of coherently moving dots, measured as the percentage of all dots presented on the screen, the so-called coherence level, controls the difficulty of the task. It is an experimental standard to also use a coherence level of 0%, where the stimulus is generated by using only randomly presented dots. The 0% coherence condition is typically used for specific experimental reasons, e.g., to control for the effect of trial-to-trial stimulus variation on neural measurements (Britten et al., 1992, 1996; Bair and Koch, 1996; Cohen and Newsome, 2009) and has been used in theoretical studies to model neural or behavioral responses when there is no net motion evidence (Wang, 2002; Wong et al., 2007; Wimmer et al., 2015). In these studies, one typically implicit assumption about 0% coherence stimuli, by way of their construction, was that the expected net motion over stimulus presentation time, i.e., the accumulated motion cues, is zero across the presentation time of the stimulus (Britten et al., 1993, 1996). When using this implicit assumption, the concrete random instantiation of a RDM stimulus in a single trial is considered uninformative about the choice made by the participant, because the stimulus on average does not contribute decision-relevant information and thus should not influence decisions.

This implicit assumption has been supported by a specific finding about the so-called choice probability. Choice probability is a measure of how strongly the firing of a single neuron can predict an animal’s decision. The finding was that the distribution of choice probabilities across neurons was comparable in response to 0% coherence RDM stimuli, both when a specific instantiation of a 0% coherence stimulus was presented repeatedly and when different 0% coherence stimuli were presented (Celebrini and Newsome, 1994; Britten et al., 1996; Cohen and Newsome, 2009). The interpretation of this observation was that the fluctuations in choices induced by the stimuli were at least not greater than those induced by internal neuronal variability. However, recent theoretical considerations and a re-analysis of data indicate that random stimulus fluctuations indeed have a measurable effect on choice probability (Wimmer et al., 2015).

Motivated by these recent findings, we tested directly whether seemingly random stimulus fluctuations as presented in 0% coherence motion in a typical RDM task does influence behavioral choices of human participants, and used a computational model to test the influence of the stimuli on choice behavior. Such a finding would show that (i) 0% coherence RDM stimuli can only serve as an approximation to a control condition, relative to conditions of coherently moving stimuli, and (ii) it may be useful to use computational models of within-trial decision evidence dynamics to better understand the mechanism of how stimulus details and expectations of the human decision maker interact, at the single trial level.

In our study, identical replicates of incoherent RDM stimuli were presented to human participants, where participants did not realize that these stimuli were repeated. Under the assumption that the stimuli provide no decision-relevant information, participants should exhibit no preference for either of the two choices across repetitions of the same 0% coherence stimulus. In contrast, we found that there are specific instantiations of 0% coherence level RDM stimuli for which, both on an individual and group level, there was a consistent choice pattern, i.e., a preference for one of the two choices. Using computational modeling, we found that these behavioral responses can be best explained by a model which is informed about the exact spatiotemporal details of the 0% coherence level stimuli. Previously, several studies have successfully used a motion energy algorithm to model the participants’ responses using information extracted from the RDM stimuli (Jazayeri and Movshon, 2006; Zylberberg et al., 2012; Insabato et al., 2014; Urai et al., 2017). Here, we used a rather simple dot-counting algorithm that extracts the time-dependent spatiotemporal stimulus features from the movement of group of individual dots in the RDM stimuli. Our results indicate that the high-performing participants used random stimulus features to make consistent decisions across repetitions of the same stimulus. In addition, for specific stimuli the amount of stimulus information used by the model correlated with the response consistency of participants. Our results suggest that if these stimuli are to be used in an experiment, one should control for the presence of decision relevant information in these stimuli. A viable alternative to controlling stimulus features is to use computational modeling to quantify and predict which information is used by participants to make decisions. Such an approach holds promise for future experiments, where computational models are used to fit behavior at the single trial level to better quantify the interactions with neuronal activity (Kiani et al., 2008; Brunton et al., 2013; Urai et al., 2017).

## Results

### Motion Discrimination Task

**FIGURE 1:**
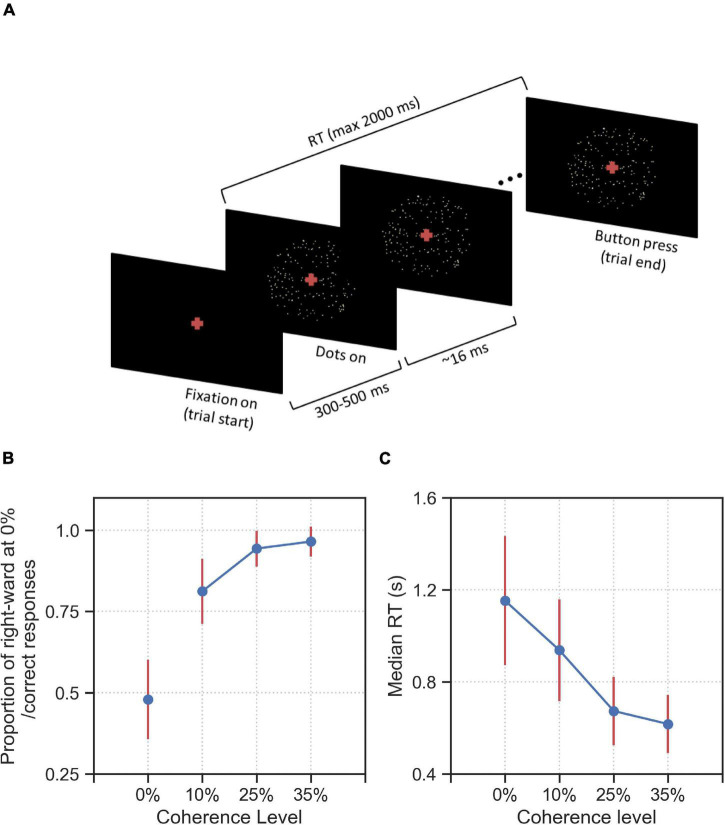
Experimental design and behavioral results on group level. **(A)** a fixation cross was shown for a variable duration between 300 and 500 ms. After this fixation period, a cloud of ∼40 dots appeared within an aperture of 12 degrees on the center of the screen. In each trial, a proportion of all dots shown, as indicated by the coherence level, were moving toward the direction (right or left) indicated by the trial-wise target alternative. The remaining dots were displaced randomly within the aperture. Dot positions were updated every 16 ms. The trial ended when the participant makes a decision (button press) or a maximum of 2,000 ms has elapsed from the onset of the first frame of dots. The task was to decide into which of two directions the dots were moving (left or right). **(B)** Proportion correct (or proportion of rightward responses for 0% coherence stimuli) and **(C)** median RT averaged over all 44 participants for the four coherence levels (0%, 10%, 25%, and 35%). Error bars indicate the standard deviation across all participants, excluding the timed-out trials.

After successfully completing a training phase (see section “Materials and Methods” for details), 44 human participants took part in a reaction-time motion discrimination (RDM) task. The main phase of the experiment consisted of 800 trials, which were identical for each participant, but were presented to each participant in a randomized order. There were four difficulty (coherence) levels: 240 trials with 0% coherence level, which were randomly interleaved among 560 trials of lower task difficulties, i.e., non-zero coherence levels (10%, 25%, and 35% coherence) with 240 trials for 10% coherence and 160 trials for 25% and 35% coherence each. For the 0% coherence level trials, we used identical replicates of fixed spatiotemporal patterns of noise (stimulus types) in the stimulus. Specifically, we used 20 identical replicates of 12 different stimulus types which were randomly interleaved between trials of higher coherence levels. These 12 stimulus types were chosen during a pilot experiment in which 32 randomly sampled stimulus types were presented to 26 participants within 0% coherence trials in different pilot runs. Of these 12 chosen stimulus types, six stimulus types were selected as they induced consistent responses in at least 50% of participants in the respective pilot run. Therefore, we estimate that one can find stimuli that induce consistent responses with a frequency of approximately 20% (6 out of 32), see section “Materials and Methods” for details of the selection of stimulus types. The other six stimulus types were selected as controls because they did not create considerable response consistency across participants in our pilot experiment. In Figure 1A we show the time-course of a single trial. A trial started with a fixation cross being shown for duration between 300 and 500 ms. Afterward, random dots appeared at the center of the screen with the fixation cross remaining through the trial. The stimulus was presented until the participant reported the decision by pressing one of two buttons (right or left) on the keyboard, but maximally for 2 s. If the participant failed to respond during this period, the trial timed-out and the next trial started with 2 s delay between trials. Participants were instructed to make accurate and fast responses, and received a monetary reward based on high overall accuracy and low average reaction times (see section “Materials and Methods” for a detailed description of stimulus construction and the experimental design).

### Behavioral Results

In Figures 1B,C we report the behavioral results of the experiment across all difficulty levels. As expected, the accuracy for non-zero coherence levels decreased for higher difficulty (Figure 1B) while RT increased (Figure 1C). The mean proportion correct (non-zero coherence level) over participants varied between 0.97 (standard deviation, *SD* = 0.04) at the 35% coherence level and 0.81 (*SD* = 0.10) at the 10% coherence level. The proportion of rightward responses at the 0% coherence level was 0.48 (*SD* = 0.12) (Figure 1B). The average median RTs varied between 616 ms (*SD* = 126 ms) at 35% coherence and 1,153 ms (*SD* = 281 ms) at 0% coherence (Figure 1C). The proportion of time-out trials was on average 0.04 (*SD* = 0.05) at 0% coherence, 0.01 (*SD* = 0.02) at 10% coherence and was below 0.01 at 25 and 35% coherence.

### Response Consistency for 0% Coherence Stimuli

**FIGURE 2:**
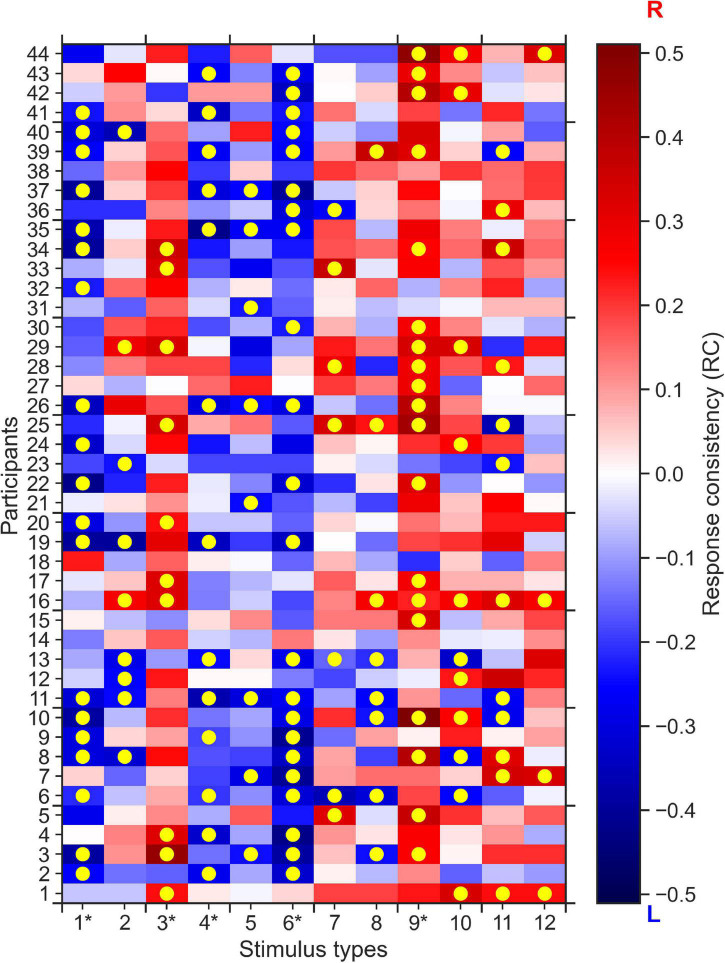
0% coherence trials: Response consistency (RC) map across 44 participants and 12 stimulus types. Positive RC values (red) report the tendency of a participant to choose the rightward direction, whereas the negative RC values (blue) report the tendency for the left direction. Yellow circles indicate significantly (*p* < 0.05, corrected for number (12) of stimulus types) consistent right (dark-red) and left (dark-blue) responses of a specific participant, across up to 20 repetitions of a specific stimulus type. One can clearly see that for stimulus types 1, 3, 4, 6, and 9 (indicated by an asterisk), most participants have mostly the same consistent responses, e.g., we see mostly red colors for stimulus type 9, i.e., the participants tend to respond right more than they do overall.

Here, we show that, in response to some specific 0% coherence stimuli, participants made behavioral choices that were consistent on the individual and group level. To do this, we first define response consistency (RC) as a measure to quantify the tendency of a participant to respond for each alternative (right or left) more than usual given a particular stimulus type. The RC values are computed as the fraction of right responses of each participant given each stimulus type minus the overall bias of the participant across all trials for the right response; a RC = 0 indicates no response consistency, i.e., no tendency for left or right responses given that stimulus type, whereas RC∈(0,1] indicates the tendency for choosing the right alternative (when RC = 1, the participants always gave a right response), and RC∈[−1,0) indicates the tendency for choosing the left response (when RC = −1, the participant always chose the left response, see section “Materials and Methods” for details of RC computation). In Figure 2 we show the map of RC values for each of the 44 participants and each of the 12 stimulus types. The higher the absolute RC value the higher the tendency of a participant to choose right (RC > 0) or left (RC < 0) alternatives for a specific stimulus. Across all stimulus types and participants, we could not find evidence for the hypothesis that RC values on average differed from 0 (two-tailed *t*-test, *p* = 0.78).

To formulate the significant response consistency of a participant given a stimulus type, we used the two-tailed binomial test. Our null hypothesis was that the participants’ expected probability of choosing right alternative given each stimulus type is equal to their overall response bias i.e., the probability of choosing the right alternative given any stimulus type. If the two-tailed binomial test with such expected probability resulted in a *p*-value smaller than 0.05, we rejected the null hypothesis and concluded that the participant has a significant consistent response toward one alternative (see section “Materials and Methods” for details). Figure 2 shows that after multiple comparison correction for the 12 stimulus types and 44 participants, more than 20% of overall responses of 44 participants to 12 stimulus types are consistent (highlighted by yellow circles).

**FIGURE 3:**
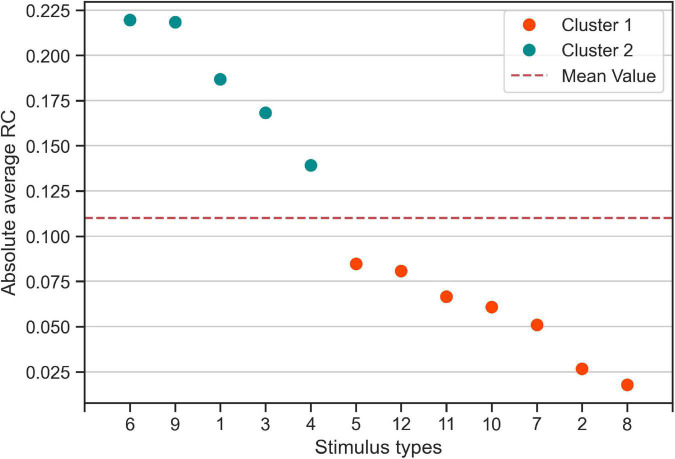
Categorization of stimulus types based on absolute average response consistency (RC) values. Using the absolute of the RCs averaged across participants, we used K-means clustering to categorize the twelve 0% coherence stimulus types according to consistency averaged over participants. Cluster 1 (Orange) contains seven stimulus types for which participants made responses that are less consistent than the average (red dashed line). Cluster 2 contains the five stimulus types to which participants responded more consistently than on average.

Furthermore, we found that for five out of the 12 stimulus types most participants tended to give (the same) consistent response, i.e., a consistent rightward response for stimulus types 3 and 9, and consistent leftward responses for stimulus types 1, 4, and 6. To quantify this difference in group-level response consistency between stimulus types more formally, we conducted k-means clustering of the absolute average RC values for each stimulus type. The k-means clustering algorithm identified two clusters of stimulus types (see Figure 3): Cluster 2 stimulus types (stimulus types 1, 3, 4, 6, and 9 identical with the consistent stimulus types identified in the analysis on the individual level) to which the participants, on the group-level, made more consistent responses than on average. Cluster 1 stimulus types consisted of the remaining stimulus types that participants responded less consistently to on average.

### Computational Modeling of Consistent Responses

Our behavioral analysis has shown that participants respond more consistently to some stimulus types than to others (Figure 2). One possible source of response consistency is the presence of specific information in the 0% coherence stimuli that participants might perceive within each trial. To investigate the underlying mechanisms of the consistent responses for the 0% coherence stimulus types, we used a computational model that accounts for the response behavior of the participants using time-dependent stimulus information (measure of random stimulus fluctuations that represent the motion toward one alternative). Further, we used Bayesian model selection to compare the model that incorporates the exact stimulus information against the one using average stimulus information.

We tested, using model comparison, whether there is a systematic relationship between the presence of such motion cues and the experimentally measured response consistency, as indicated by Figure 2. We used the behavioral data (both choices and reaction times). The first model was a discrete time version of the well-established drift diffusion model (DDM) (Ratcliff, 1978; Ratcliff and McKoon, 2008). The second model was an adapted DDM version where the model was informed about the trial-wise coherent motion cues in the sensory stimuli. To measure coherent motion cues, we counted dots that indicated a coherent movement in one of the two motion directions within a small time window. In the following, we call this second model the Exact Input Model (EXaM) (see section “Materials and Methods” for detailed description of the dot count measure methods and the models). If we find that this second model explains the observed behavioral data better, according to Bayesian model selection, this is further evidence that the exact dot stimuli are actually used by participants when making their decisions.

We estimated the parameters of the DDM and the EXaM using a Bayesian inference technique called Expectation propagation-Approximate Bayesian Computation (EP-ABC) (Barthelmé and Chopin, 2013). We used 10 free parameters for both models, similar to the standard DDM but adapted to the present experiment: The (1) mean scale parameter ${\bar{s\,c}}_{1}$ for the subset of trials in which Cluster 1 stimulus types were presented and (2) the mean scale parameter ${\bar{s\,c}}_{2}$ for the trials of Cluster 2 stimulus types (cf. Figure 3). The mean scale parameters represent the average proportion of the evidence adapted from the trial-wise stimulus information. We fitted two mean scale parameters to test whether there are any motion signals in the Cluster 2 stimulus types, relative to Cluster 1, that cause consistent responses across participants, (3) the scale parameter standard deviation σ_*sc*_, (4) the bound parameter *B*, which is the threshold of accumulated evidence to commit to a decision, (5) The mean ${\bar{z}}_{0}$ and (6) standard deviation *s_Z_* of participant-specific bias for an alternative, (7) the mean ${\bar{T}}_{n\,d}$ and (8) the standard deviation *s*_*t*_of the non-decision time i.e., the portion of the RT which is different from the decision process (i.e., perceptual encoding, motor preparation), (9) lapse probability π_*l*_: the proportion of trials leading to a random response, and (10) timed-out lapse probability π_*to*_: the proportion of timed-out trials within lapse trials. We inferred the parameters of the DDM and the EXaM from the behavioral data (choice and response time for each single trial) separately for each participant and for each of the four coherence levels (see section “Materials and Methods” for details).

#### Model Comparison (Exact Input Model vs. Drift Diffusion Model)

Here, we focus on a model comparison question: does equipping a DDM-like model with the motion cues makes it a better model to explain the behavioral data? In other words, is the EXaM a better model to explain the participants’ responses at the 0% coherence level than the DDM? To address this question, we formally compared the two models using a two-way Bayesian model comparison (Penny et al., 2010).

**FIGURE 4:**
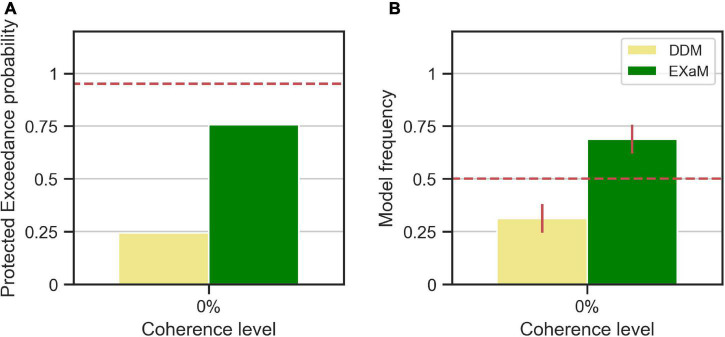
Results of random-effects Bayesian model comparison between DDM and EXaM for 0% coherence across 44 participants, **(A)** Protected exceedance probability (probability that a model is the best model for all participants). The red dashed line indicates very strong evidence for a model (0.95). **(B)** Model frequency, i.e., the probability that a randomly selected participant’s behavior is best explained by the specific model. The red dashed line represents chance level and error bars indicate the standard deviation of the estimated model frequencies.

We computed the protected exceedance probabilities and model frequencies (i.e., posterior model probabilities) (Stephan et al., 2009; Rigoux et al., 2014) based on the model evidences estimated by EP-ABC (see Figure 4). We found evidence that the EXaM explains the responses of the participants better than the DDM. The protected exceedance probability (Figure 4A), i.e., the belief that the EXaM is across all participants a better model of the data than the DDM, was around 76%. The model frequency (Figure 4B), i.e., the probability that the EXaM generated the data for any randomly selected participant, was around 69%.

As a control analysis, we also conducted the same model comparison for the behavioral data of higher coherence levels (10%, 25%, 35% coherence) (Supplementary Figure 1). As expected, at higher coherence levels, the EXaM was not a better model than the DDM in explaining the behavioral data.

#### Differences in Parameter Values (Exact Input Model vs. Drift Diffusion Model)

To understand which model parameters cause the EXaM to outperform the DDM in explaining the behavioral data, we computed the means of the posterior parameter distributions (see Table 1 for 0% coherence level, Supplementary Table 1 for higher coherence levels) for the two models. We hypothesized different mean scale parameter estimates for Cluster 2 stimulus types (${\bar{s\,c}}_{2})$ for the EXaM as compared to the DDM, but not for Cluster 1 stimulus types (${\bar{s\,c}}_{1}).$ When comparing posterior means, we found two main differences: the EXaM, in comparison to the DDM, had (i) a higher mean scale parameter ${\bar{s\,c}}_{2}$ for Cluster 2 stimulus types, and (ii) a higher (more neutral) mean bias parameter, ${\bar{z}}_{0}$. Also, the EXaM had significantly higher posterior mean estimates of the variability of the scale parameter σ_*sc*_ compared to the DDM. However, the effect size is rather small.

**TABLE 1:** Mean of the average posterior parameter distributions for the DDM and EXaM for 0% coherence across 44 participants.

Parameters	DDM	EXaM
	0%	0%
${\bar{\text{sc}}}_{1}$	0.02 (0.005)	0.01 (0.003)
${\bar{\text{sc}}}_{2}$	0.01 (0.003)	0.037** (0.005)
σ_**sc**_	0.05 (0.001)	0.053** (0.001)
**B**	0.05 (0.002)	0.05 (0.002)
${\bar{\text{z}}}_{0}$	−0.13 (0.030)	−0.025** (0.028)
**s_Z_**	0.02 (0.000)	0.02 (0.000)
${\bar{\text{T}}}_{\mathrm{nd}}$	0.81 (0.049)	0.79 (0.046)
**s_t_**	0.63 (0.054)	0.59 (0.046)
π**_l_**	0.07 (0.016)	0.06 (0.013)
π**_to_**	0.34 (0.023)	0.34 (0.024)

Shown are the means over participants and the corresponding standard error in parentheses. The mean bias (${\bar{z}}_{0})$ and variability of bias (s_Z_) are both in terms of proportions of the bound (B) (ranging from −1 to 1). A mean bias of 1 indicates a complete bias toward the right choice and −1 a complete bias toward the left one. Asterisks indicate a significant difference between DDM and EXaM parameters for each condition (**p < 0.01, based on a paired t-test over 44 participants). See description of the EXaM in section “Materials and Methods” for the meaning of parameters.

### Source of Response Consistency

We have shown that the participants made consistent responses for Cluster 2 stimulus types and that the EXaM which was informed about the precise spatiotemporal stimulus features explained the behavioral data better than a DDM-equivalent model. These findings indicate that the participants’ consistent responses to 0% coherence stimulus types are driven by motion cues in the sensory stimuli. If this is the case, we would expect that this effect can be even more strongly observed if the participants are motivated and attentive to the task, i.e., to the fine spatiotemporal details of the stimuli.

To test this, we contrasted (i) the scale parameter estimates as determined by the EXaM for high and low-performing participants. To classify participants’ performance, we used their overall task performance in the non-zero coherence levels, i.e., proportion correct in 10%, 25%, and 35% coherence levels. We defined two groups of participants with (1) the 25% low-performing participants, and (2) the 25% high-performing participants (both groups have 11 participants), see Supplementary Figure 2. As this was the selection criterion, the high-performing participants had a higher proportion correct in all non-zero coherence levels (Supplementary Figure 2A, *t*-test, *p* < 0.001). Interestingly, the high-performing participants also had significantly faster median RTs at higher coherence levels (25% and 35% coherence levels) (Supplementary Figure 2B, *t*-test, *p* < 0.01). This indicates that these participants were indeed more motivated to do the task, as opposed to just increase accuracy by sampling for a longer decision time. Note that, at the 0% coherence level, we cannot compare the proportion correct between high- and low-performing participants because the correct alternative is undefined. Strikingly, when using a regressing analysis for the non-zero coherence level, we found a correlation between proportion correct and the average absolute value of response consistency in 0% coherence trials (Supplementary Figure 3, linear regression Eq. 2, *R* = 0.35, *p* < 0.05). This means that the more accurate, on average, the participants are in their decision in non-zero coherence trials, the more consistent they are, on average, in response to 0% coherence level stimulus types.

**FIGURE 5:**
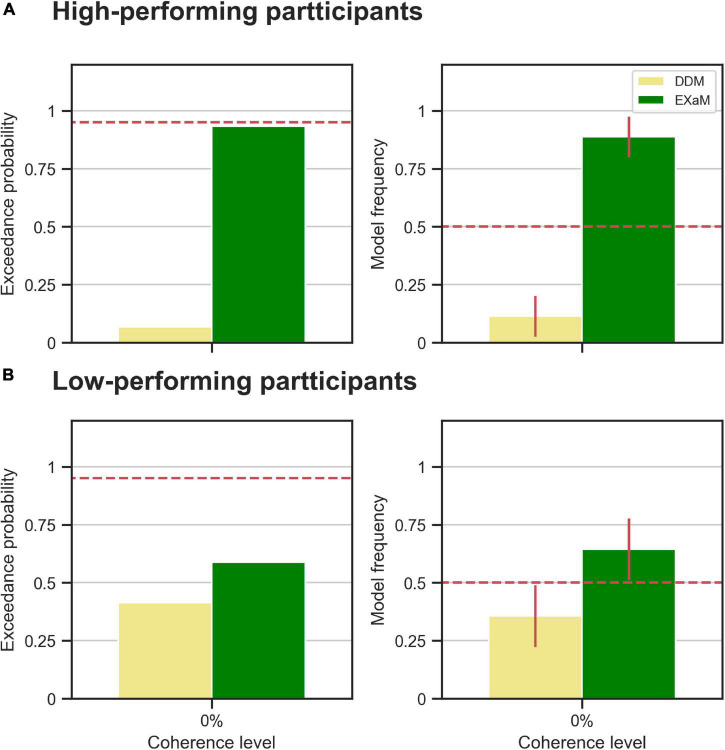
Results of random-effects Bayesian model comparison for zero % coherence level across two groups of participants; **(A)** the observed model comparison results in Figure 4 are actually driven by the high-performing participants. The left plot shows the protected exceedance probability (probability that a model is the best model for all participants). The red dashed line indicates very strong evidence for a model (0.95). The right plot shows the model frequency for model comparison between DDM and EXaM. The model frequency is the probability that the behavior of a randomly selected participant is best explained by a specific model, among the compared models. The red dashed line represents chance level and error bars indicate the standard deviation of the estimated model frequencies. **(B)** The same plots as in A for the low-performing participants.

We also compared models between the high- and low-performing participants, i.e., is the EXaM also the better model, compared to the DDM, in explaining the responses of these two groups of participants? We compared the DDM and EXaM using 2-way Bayesian model comparison for both groups of participants (high- and low-performing) (Figure 5). We found strong evidence that the EXaM better explains the responses of high-performing participants but not so for the low-performing participants. In high-performing participants, the protected exceedance probability was over 93% for EXaM and the model frequency was 88% (Figure 5A). In contrast, for low-performing participants, the protected exceedance probability was 59% for the EXaM and the model frequency was 64% (Figure 5B). These results suggest that the EXaM is the better model (Figure 4) mainly because of the high-performing participants.

#### Differences in Exact Input Model Parameter Values (High- and Low-Performing Participants)

Is there any difference between the estimated parameters of the EXaM for the participants who are high-performing in the RDM task compared to participants who are not? There was no significant difference between the two groups of participants for the mean scale values for both Cluster 1 and Cluster 2 stimulus types, but we found that the high-performing participants had a higher mean scale value compared to the low-performing participants for Cluster 2 stimulus types (${\bar{s\,c}}_{2}$) (see Table 2). This difference was, however, not statistically significant (*p* = 0.068) which may be explained by the rather low number of participants (*N* = 11 for both high- and low-performing participants). These results suggest that the model uses the mean scale parameter to explain the behavioral data for the high-performing participants for Cluster 2 stimulus types.

**TABLE 2:** Mean of average posterior parameter distributions of EXaM for the high- and low-performing of participants.

Parameters	EXaM (High-performing participants)	EXaM (Low-performing participants)
	0%	0%
${\bar{\text{sc}}}_{1}$	0.026 (0.009)	0.012 (0.003)
${\bar{\text{sc}}}_{2}$	0.064 (0.018)	0.024 (0.003)
σ**_sc_**	0.055 (0.002)	0.053 (0.002)
**B**	0.054 (0.004)	0.060 (0.004)
${\bar{\text{z}}}_{0}$	−0.067 (0.058)	0.052 (0.058)
**s_Z_**	0.021 (0.000)	0.022 (0.001)
${\bar{\text{T}}}_{\mathrm{nd}}$	0.904 (0.101)	0.681 (0.070)
**s_t_**	0.658 (0.081)	0.582 (0.095)
π**_l_**	0.089 (0.031)	0.063 (0.022)
π**_to_**	0.307 (0.060)	0.357 (0.048)

*Format as in Table 1, see also description of the EXaM in section “Materials and Methods” for the meaning of parameters.*

#### The Relationship Between Response Consistency and Scale Parameter

**FIGURE 6:**
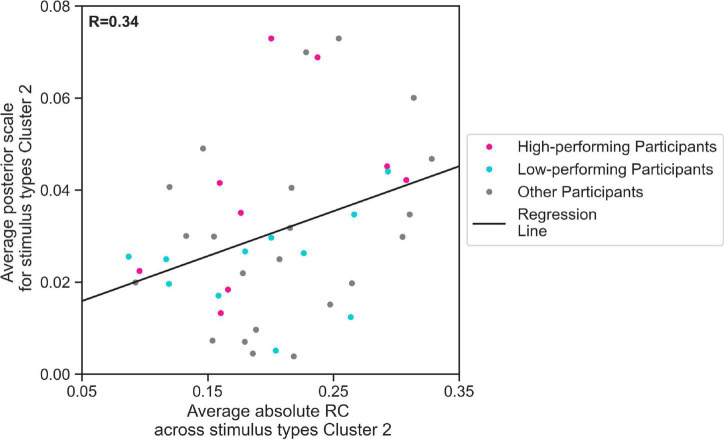
The relationship between zero % coherence response consistency and average posterior estimates of the EXaM’s scale parameter. The average posterior scale estimate for Cluster 2 stimulus types (${\bar{\text{sc}}}_{2}$) is plotted as a function of average absolute RC across Cluster 2 stimulus types. The regression line shows a positive correlation between two variables (linear regression Eq. 2, *R* = 0.34, *p* < 0.05*).* The data from two participants was excluded as their ${\bar{\text{sc}}}_{2}$ values were outliers.

We further evaluated, using regression analysis, whether there was a linear relationship between the estimated mean scale values and the average response consistency, over all 44 participants. We conducted our regression analysis for ${\bar{s\,c}}_{2}$ and average absolute RC across trials containing Cluster 2 stimulus types. Indeed, our results show that the estimated ${\bar{s\,c}}_{2}$ values for 44 participants increased with the average absolute RC across Cluster 2 stimulus types (Figure 6, linear regression Eq. 2, *R* = 0.34, *p* < 0.05). This means that the more consistent the participants in their responses to Cluster 2 stimulus types, the higher the ${\bar{s\,c}}_{2}$ parameter the EXaM used to explain the behavioral data. We have found a considerable difference between the slope of the regression line representing the relationship between estimated ${\bar{s\,c}}_{2}$ values and average absolute RC across Cluster 2 stimulus types for high-performing (0.146) comparing to the similar slope for the low-performing participants (0.048). Although the regression analysis did not show a significant relationship between the above-mentioned variables for neither high-performing participants (*R* = 0.48, *p* = 0.2) nor low-performing participants (*R* = 0.31, *p* = 0.4), but this can be explained by small sample size used for this analysis (*N* = 11).

## Discussion

We presented evidence of a consistent choice pattern in humans in response to identical replicates of 0% coherence random-dot motion stimulus (RDM). Specifically, we have quantified the preference of human choices across repetition of the same 0% coherence stimulus using a simple statistical measure. To formally model these consistent choices, we have extended the standard drift-diffusion model to incorporate the exact time-dependent stimulus features, the exact input model. Our model comparison shows that the exact input model can better explain the consistent responses of the participants to specific instantiations of 0% coherence level RDM stimuli compared to a standard drift-diffusion model. This means that participants use stimulus information of the 0% coherence stimuli to make consistent choices. We found the difference between the two models much more pronounced for high-performing participants, which indicates that these participants use more of the information shown in the sensory stimuli, as compared to low-performing participants. In addition, the amount of stimulus information that the model uses to explain the consistent responses of high-performing participants was positively correlated with the behavioral consistency of the participants.

One important implication of our experimental findings is related to the use of 0% coherence RDM stimuli in the analysis of the role of sensory stimuli in neural and behavioral measurements such as choice probability (Britten et al., 1992, 1996; Bair and Koch, 1996; Cohen and Newsome, 2009). In these experimental studies, the 0% coherence RDM stimuli were typically used to control for trial-to-trial stimulus variation in the observed effects of choice probability. For example, it has been implicitly assumed before that since the expected net motion of 0% coherence stimuli over the presentation time is zero, this type of stimuli is “identical for any particular direction,” therefore the observed correlation between neural and behavioral measurements (choice probability) is unlikely to be due to trial-to-trial variability of the stimulus (Britten et al., 1992). This assumption was only challenged recently by Wimmer et al. (2015) which have demonstrated the effect of random stimulus fluctuations on the measurements of choice probability. We have conducted a dedicated investigation of the role of time-dependent stimulus information in human responses to 0% coherence RDM stimuli. By systemically studying the effect of stimulus types on behavioral choices, we provide another piece of evidence supporting the hypothesis that some specific 0% RDM stimuli types do have an effect on the participants’ decisions on the group-level and contain decision-relevant information. Therefore, random motion and similar stimuli should be controlled for such decision-relevant information when they are used in an experimental study e.g., in the measurement of choice probability. This type of single-trial analysis of the behavior (Kiani et al., 2008; Brunton et al., 2013; Urai et al., 2017) can also be promising to quantify the relationship of the behavior to neural measurements.

To better understand the possible reasons of observing response consistency in the trials with Cluster 2 stimulus types (Figure 3), we have considered several sources and potential confounding factors: (i) randomness in participants’ responses due to the difficulty of the RDM task in 0% coherence trials, (ii) serial dependence of participant responses in trials with 0% coherence stimuli, (iii) general bias of the participants for an alternative, (iv) error in statistical inference for determining consistency, (v) presence of particular motion patterns in Cluster 2 stimulus types that makes the average participant to respond consistently across repetition of the same stimulus type. First, we made sure, through a training phase, that only participants that had less than 7% of the training phase trials as timed-out trials and were more accurate than 85% in 25% and 90% in 35% coherence level, attended the main phase of the experiment. Also, in the experimental design we made sure that there are a limited number (max 5) of consecutive hard trials (0% and 10%). These constraints ensured that the responses of the participants to the 0% coherence stimuli are not “random.” Also, all stimulus types used in our experiment (Cluster 1 and Cluster 2 stimulus types) were chosen after careful evaluation of 32 randomly sampled stimulus types within the pilot phase for the ability to induce consistent responses across a wide group of participants (see section “Materials and Methods” for more details). Second, the random distribution of the stimulus types within the trials during the course of the experiments makes it unlikely that serial dependence of participant responses of the trials with 0% coherence (consequently Cluster 2 stimulus types) stimulus be a source of the observed effect in response consistency (Figure 2). The reason is that all 0% coherence trials were randomly interleaved between trials of higher coherence levels in a way that there were no 4 consecutive trials in which the same stimulus type is presented. Third, in the statistical test used for determining response consistency, we corrected the response consistency for a general bias of participant for one alternative to ensure that the general bias does not affect significance response consistency values. Fourth, we used multiple comparison correction to ensure the reliability of the significance of our response consistency measurements. By ruling out all of the previous possibilities, we conclude that the remaining explanation for consistent responses of the participants is that the participants use the motions patterns inside Cluster 2 stimulus types to make consistent responses. We have found strong evidence that the responses of high-performing participants are explained better by the exact input models that use the stimulus features (dot counts) (Figures 5, 6). As the stimulus features we used in our analysis represent the net rightward motion of all dots within the stimulus aperture through time, therefore, we conclude that there are some motion patterns in these specific 0% coherence stimulus types that the participants use to respond consistently to 0% coherence stimuli.

We found that the EXaM, compared to the DDM, uses a significantly more neutral mean bias parameter, ${\bar{z}}_{0}$, for Cluster 2 stimulus types (see Table 1). This finding offers another piece of evidence that the EXaM provides for a better model and explanation of the participants’ consistent responses to 0% coherence stimuli: As Figure 2 shows, three out of five of Cluster 2 stimulus types contain signals that induce consistent responses among the participants indicating leftward motion in the 0% coherence stimuli. The DDM, in the absence of stimulus informations, on average uses a negative mean bias parameter to explain the majority of leftward consistent responses to Cluster 2 stimulus types across the participants. In contrast, the EXaM is informed about motion patterns in the stimuli and therefore does not use the mean bias parameter to explain the participants’ responses.

In the present study, we aimed at showing that one should expect that 0% coherence stimuli contain some cues that has a consistent effect on the choices of human participants. In our study, we did not assess how probable it is to stochastically sample a stimulus that does contain small cues that the participants are sensitive to. However, we assume that this is not too uncommon because we had no difficulty in identifying six of these stimuli in a random sample of 32 stimuli.

One open and profound question is how exactly motion cues during 0%-coherence trials are used dynamically to make a decision. In the proposed model, all motion cues measured by the dot counting algorithm are weighted identically by some scaling factor to explain both choices and reaction times. Although this model appears intuitive at first glance, it can only be a first approximation of some more complex decision making machinery. In the exact input model, we assume that the brain uses a different scale parameter between the two stimulus types. It is an open question how the brain can adjust its scale parameter for an ongoing trial, if it is unknown at the beginning of the trial of what type the current stimulus is. We speculate that the brain can quickly adapt its expectation given an initial period of an ongoing trial, see also (Wimmer et al., 2015) for a similar perspective. This initial period may give the brain the time to adjust its expectation about the amount of movement it will encounter during the remainder of the trial. This dynamic interaction between sensory data and a potentially higher level parameterization set would be most naturally expressed in a predictive coding framework (Friston and Kiebel, 2009). This view also resonates well with findings of Wimmer et al. (2015), who proposed to portrait decision making as a dynamic interplay between bottom-up and top-down interactions.

The application of time-dependent stimulus features, e.g., motion energy, has been used before within the context of bounded accumulation models similar to DDM (Jazayeri and Movshon, 2006; Kiani et al., 2008; Zylberberg et al., 2012; Insabato et al., 2014). In the present study, we have used a rather simple dot-counting algorithm to quantify the spatiotemporal stimulus features based on RDM stimuli that captures the effect of individual dot movements with high precision. However, the exact characteristics of the motion patterns that lead to response consistency in 0% coherence stimuli are unknown to us, because the dot counts are a global spatial measure that are simply the sum of net rightward motions over the whole stimulus aperture. It may be possible in future studies to specify the motion patterns in the above-mentioned 0% coherence stimulus types by using more advanced measurements of dot counts by tracking the eye movements of the participants and computing the stimulus features only in attention focus areas within the stimulus aperture. Using model comparison, one may find an attention focus area that can maximize the ability of the exact input model in explaining the behavioral responses of the participants. The use of eye-tracking can also help to (i) measure the attentiveness of the participants during the course of the experiment and use it as an extra feature for the exact input model, and (ii) identifying the characteristics of the motion patterns that can induce response consistency facilitates the process of finding more similar stimulus types for future experimental studies.

The dot counts capture the direction-wise movement of the dots within the stimulus aperture at every time point without spatiotemporal filtering, in contrast to the motion energy algorithm (Adelson and Bergen, 1985; Shadlen and Carney, 1986). This leads to simplicity of capturing individual dot movements which we consider essential for interpreting the model-based analysis of responses consistency with incoherent RDM stimuli. The simplicity of the dot counting algorithm makes it an intuitive option for computing the stimulus feature from random-dot motion stimuli. On the other hand, one limitation of the dot counting algorithm might be that it does not differentiate between directed movements with different angles. For example, in every time step, it will detect the movement of the dot within a pre-defined square area to the right of the position of the dot in the previous time step, as a rightward movement (see section “Materials and Methods” for details of computation of dot counts) but does not add graded evidence depending on the computed angle of the movement.

The modeling approach used in this study extended the line of work on including the spatiotemporal details of stimulus into the model-based analyses of the behavior (Kiani et al., 2008; Brunton et al., 2013; Insabato et al., 2014; Park et al., 2016; Fard et al., 2017). The exact input model in the present study has enabled the exact input modeling approach introduced in the previous studies (Park et al., 2016; Fard et al., 2017) to account for consistent responses of the participants to 0% coherence stimuli. The exact input model in the current study uses a similar evidence accumulation process comparing to the DDM (using the scale parameter and dot counts) and this enables the exact input modeling approach to be able to account for the behavioral data from random dot motion discrimination task comparing to the previous exact input modeling approaches (Park et al., 2016; Fard et al., 2017). Furthermore, the application of scale parameter in combination with dot counts in the EXaM enables it to account for a time-dependent evidence accumulation rate that can be multiplied with the stimulus features and this creates a better explanatory power for our behavioral data comparing to DDM’s drift rate that is constant within a trial (Ratcliff, 1978; Ratcliff and McKoon, 2008).

## Materials and Methods

### Participants

60 healthy humans were recruited to participate in the study. All participants were right-handed, had normal or corrected eyesight and without any symptoms of color-blindness or deficiency in stereopsis. The participants gave informed written consent. The experimental procedure was approved and carried out in accordance with the guidelines by the ethics committee of the Dresden University of Technology. Out of 60 participants, 45 passed the training phase according to the criteria related to their performance in the task (see below). One participant was excluded from further analysis because in the main phase the performance of the participant dropped below the threshold indicated by completion criteria of the training phase (see Experimental Procedures below) i.e., the participant’s proportion correct was below 85% in trials with 25% and below 90% in the trials with 35% coherence level stimulus. Therefore, 44 participants (mean age 24.9 years, 25 females) were included in our analysis. All of the timed-out trials were excluded from the behavioral analysis.

### Experimental Stimuli

Visual stimuli were presented using MATLAB Psychtoolbox^1^ on a 1920 * 1080 LED monitor (refresh rate: 60 Hz). Participants were seated in a chair ∼60 cm from the monitor. The random dot motion stimuli appeared in a 12°-diameter aperture at the center of the screen. The aperture size was chosen in accordance with previous studies (Urai et al., 2017) to create a trade-off between eye movements and maintain the participants’ fixation within a limited area. The dots were white 6 × 6 pixels square on a black background resulting in an average dot density of 16.7 dots/degree^2^/s. The random-dot motion stimulus was created from three independent sets of interleaved dots each being presented repeatedly every three frames (50 ms). Between every two presentations of each set of dots, a fraction of dots were moving coherently according to the direction of the motion assigned to the trial (right or left), while the rest of the dots were displaced randomly. The fraction of coherently moving dots was determined by the stimulus strength (coherence level) associated with each trial. The motion stimulus is described in detail previously (Shadlen and Newsome, 2001; Roitman and Shadlen, 2002; Kiani et al., 2013; Urai et al., 2017).

### Experimental Procedure

During the training phase, we ensured that only participants with high amount of attention and task performance are able to attend the main phase of the experiment. This was done by assessing the performance of the participants during the training phase and only allowing the ones who meet the minimum performance criteria to proceed to the main phase. Participants underwent thorough training before performing the main experiment. Initially, the participant was trained for a minimum of 200 trials (∼ 15 min). The performance of the participant (RT, choice accuracy, and number of timed-out trials) was evaluated for trials 101–200. The participant could proceed to the main phase of the experiment upon meeting all of the following performance criteria: whether the proportion of correct decisions was (1) above 85% (approx. max 3 errors) in the trials with 25% and (2) above 90% (approx. max 2 errors) in the trials with 35% coherence level stimulus, and (3) whether the proportion of timed-out responses was below 7%. If the participant did not meet the aforementioned criteria in the first attempt of the training, the experimenter allowed a second attempt of the training for maximally another 100 trials (∼7 min). The participant could still proceed to the main phase of the experiment if they passed the second attempt to the training phase according to the criteria mentioned above (1–3). Otherwise, the participant was prevented from proceeding to the main phase of the experiment.

The main phase consisted of 800 trials and took around 50 min, excluding the time dedicated for 3 breaks (given at every 200 trials). Participants were compensated at minimum rate of 10 Euros. In order to promote motivation and maintain attention, participants were rewarded for high accuracy and adequate reaction time. Depending on the performance of each participant, a bonus was given in the range from 0 to 5 euros. After the experiment, participants filled out a short questionnaire about the experiment, to check how well they have maintained their attention throughout the study.

In all phases of the experiment, four different coherence levels were used for the RDM stimulus: 0% (incoherent, the hardest), 10%, 25%, and 35% (the easiest) coherence. A fixed number of trials was dedicated for each coherence level: 240 trials each for 0% and 10% coherence (60 trials during the training phase), and 160 trials each for 25% and 35% coherence (40 trials during the training phase). In 0% coherence trials, we used 20 identical replicates of each of 12 fixed spatiotemporal patterns of noise (frozen noise) in the RDM stimuli (see below). We also call these 0% coherence frozen noise stimuli instantiations the “stimulus types.” These trials were randomly interleaved between the trials containing RDM stimuli of higher coherence levels. The design of the experiment was identical across all participants. For each participant, the trials were pseudo-randomly ordered according to two rules: (i) The trials containing the 0% and 10% coherence stimulus (harder trials) were randomly interleaved between trials containing 25% and 35% coherence stimulus (easier trials) (similar to Britten et al., 1996) such that there were no 6 consecutive hard trials. This was to prevent the participants from losing motivation during the experiment. (ii) There were no 4 consecutive trials containing the same 0% coherence stimulus type. This was to prevent the participants from finding out that identical replicates of stimuli were presented to them.

A trial started with a fixation cross appearing in the center of the screen for 300–500 ms (truncated exponential) over which the participants were asked to maintain fixation. Then the RDM stimulus appeared on the screen in an aperture with 12 deg diameter around the center of the screen (location of fixation cross). Participants were free to fixate at any position within the stimulus aperture. The stimulus was presented until the participant reported the decision by pressing one of two buttons using the index finger of the right hand (on the key “M”) or the left hand (on the key “Z”) over the keyboard. The participants could respond maximally for 2 s. If the participant failed to respond during this period, the trial timed-out and the next trial started with 2 s delay between trial (see Figure 1). In the training phase a feedback of the participants’ performance is provided to them after the trial was concluded (correct, incorrect, and timed-out). In the main phase the feedback (timed-out) was only given in case the participant failed to respond within the allotted time.

### Sampling Stimulus Types

The values of time-dependent dot positions in our RDM stimulus were initialized and updated using a pseudo-random number generator (except for coherently moving dots in each frame). For each stimulus type, the pseudo-random number generator was seeded by a specific value to generate a fixed time-dependent pattern of dot positions across all frames of a trial. Therefore, the random seed value solely determined a stimulus type in 0% coherence level.

During our pilot study, we tested 32 different stimulus types in 3 three pilot runs with 26 pilot participants separate from the participants in the main study to select suitable stimulus types for our experiment. We chose 12 different stimulus types for our 0% coherence stimuli: (i) six stimulus types were chosen for their ability to induce consistent responses in most participants. Specifically, for each of these stimulus types at least 50% of participants on a particular pilot run exhibited a proportion of left choices that significantly differed from chance (see below for statistical procedures) indicating that these participants responded consistently left or right to this stimulus type. (ii) three stimulus types were chosen as the stimulus types to which the participants responded to strong evidence in the stimulus toward one alternative within a 150–400 ms, (iii) three other stimulus types did not meet any of the criteria stated in (i) or (ii) and were chosen as baseline presumably reflecting random response behavior of the participants.

### Identifying Consistent Responses

The performance of participants in perceptual decision making tasks is usually quantified by proportion correct (accuracy) which stands for the proportion of trials that the direction indicated by participant’s response (decision) matches the actual direction of coherent dot movements (correct alternative). In 0% coherence conditions, however, the correct alternative is undefined. On the other hand, Participants may have a general tendency (general response bias) for choosing one alternative more often than the other one during the course of the experiment. Note that by a consistent response, we imply that among a set of decisions a participant makes based on identical replicates of a stimulus type, one alternative is chosen consistently.

We computed the response consistency (RC) for each stimulus type and participant as the fraction of right responses among all non-timed-out responses of the stimulus type (maximum 20) minus the overall fraction of right responses across all trials of all coherence levels of that participant (rightward response bias). Theoretically, RC vary in the range of [−1,1]: with respect to the participant’s general response bias. *RC=0* indicates equal tendencies for right and left alternatives for that stimulus type, *RC* ∈ (0,1] indicates a tendency of the participant to choose the right alternative and *RC* ∈ [−1,0) indicates a tendency of the participant to choose the left alternative more often than usual. The higher the magnitude of *RC*, the stronger the participant’s tendency to respond consistently Right (RC>0) or left (RC<0).

**FIGURE 7:**
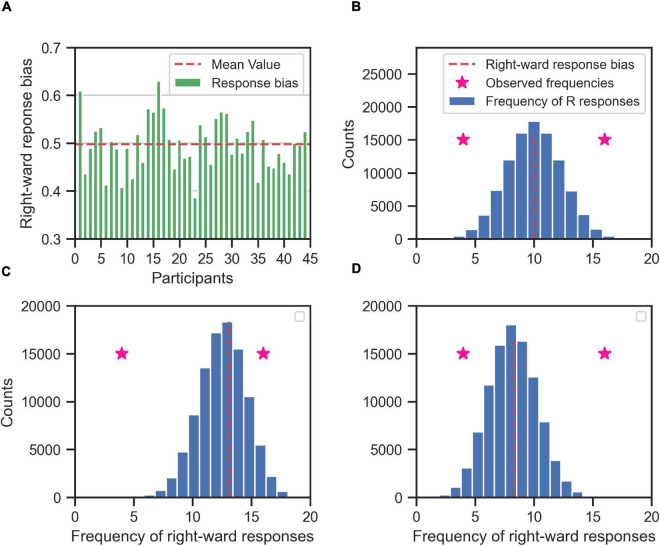
Determining the consistent responses given the response bias (right) of the participants; the rightward responses bias for each participant **(A)** is used to create the null hypothesis distribution of frequency of rightward responses (blue histogram) for each participant **(B–D, for three exemplary participants)**. The null hypothesis distribution (the blue histogram) is binomial distribution centered on the rightward response bias of the participant (red dashed line). If the participant is in general unbiased toward right or left alternatives, then the null hypothesis distribution is centered around 10 (as in **B**), i.e., half of the maximum number of responses for each stimulus type (20). If the participant is in general biased toward right **(C)** or left **(D)** the mean value of the null hypothesis is shifted toward 20 or 0, respectively. The null hypothesis distribution is used to determine the whether the observed frequency of rightward responses of the participant to a specific stimulus type (two exemplary pink stars for two different stimulus types) is consistent. If an observed frequency is extreme enough w.r.t. to mean of the null hypothesis distribution, then the response of the participant to that stimulus type is consistent. For example, the observed rightward frequency of 16 is consistent in **(B,D)**, but not in **(C)**. Likewise, the observed rightward frequency of 4 is consistent in **(B,C)**, but not in **(D)**.

To identify the consistent responses for each stimulus types on the individual level, we used the binomial test for determining which RC values were far enough from zero to state with certainty that the underlying participant responses to a stimulus type were consistent toward one alternative. In our experimental task, we had two alternatives and thus two categories of decisions: right or left. Our null hypothesis (*H_0_*) was that participants were responding equally frequently to right and left after accounting for their overall response bias. The alternative (*H_1_*) was that the participants were responding more frequently to either left or right. Since each RC value was based on the frequency of a binary response variable, we modeled the null hypothesis with a binomial distribution over the number of right responses among the 20 repetitions of a stimulus type. To account for the overall response bias of a participant (Figure 7A) we set the probability of responding right of the binomial distribution to the corresponding overall response bias of the participant computed over all trials of the main experiment (Figure 7B). To determine whether an observed number of right responses indicated that the participant responded consistently to a certain stimulus type, we computed a two-tailed *p*-value as the probability according to the modeled binomial distribution that the number of right responses could be more extreme relative to the mean than the actually observed number (Figures 7C,D). This procedure simultaneously tested for left and right consistent responses, because we summed the probability mass across number of right responses smaller and larger than their mean and with a distance larger than that for the observed number of right response.

In order to correct for the multiple comparisons, we used the false discovery rate method (Benjamini and Hochberg, 1995) over the total number of 528 hypothesis tests conducted for 44 participants and 12 stimulus types with alpha-level of 0.05.

### K-Means Clustering

To conduct the clustering over average absolute response consistency values for different stimulus types, we used the k-means clustering method (MacQueen, 1967). The k-means clustering algorithm computes the sum of point-to-cluster-centroid distances within each cluster and minimizes the sum of this quantity over all clusters. We used the Python implementation of k-means clustering using the scikit-learn toolbox^2^.

### Computing Stimulus Information Using Dot Counting Algorithm

**FIGURE 8:**
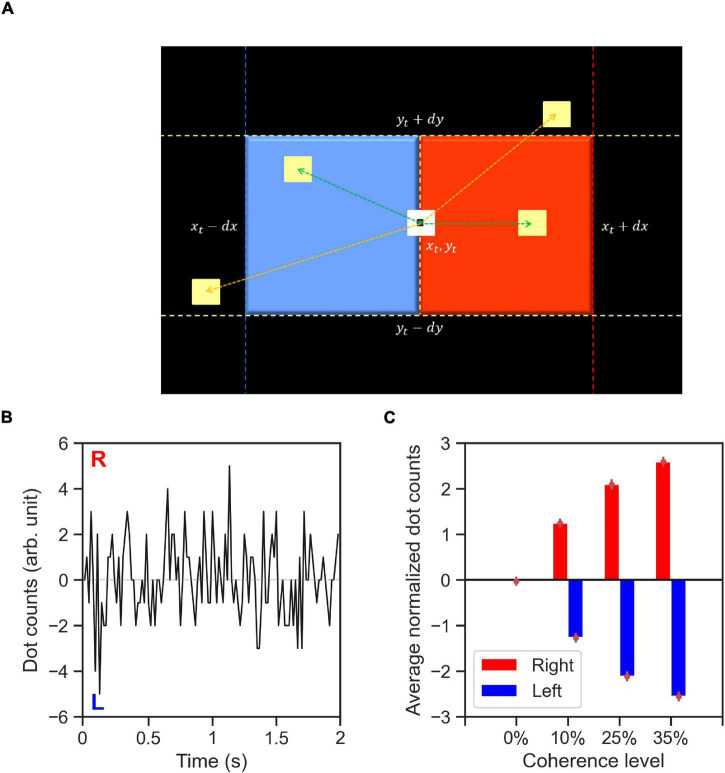
The dot counting algorithm and stimulus features. **(A)** Schematic of dot counting algorithm: the algorithm iterates through every dot within the stimulus aperture at every time-step, *t*. If within a time period of 50 ms (up to three frames) after *t* there exists a dot within a square area to the right of the current dot (red area), the dot movement is rightward (green arrow to a yellow dot within the red area) and the dot counts (DC) for the respective time-step, *dc*(*t*), is incremented. Likewise, if within the similar time period there exists a dot within the area to the left of the current dot (blue area), the dot movement is leftward (indicated by green arrow to the yellow dot within the blue area) and the *dc*(*t*) is decremented. The dot movements outside of the red and blue areas (indicated by orange arrows) dot not contribute to the value of *dc*(*t*) for the dot currently being considered, **(B)** a representative example time-course of the computed DC for a single trial with 0% coherence stimulus type 6. The labels R and L indicate the directions encoded by the DC features. **(C)** Average normalized DC values shown as a function of coherence level and trial-wise direction of correct alternative. The normalized DC values are computed as the DC values divided by the standard deviation of absolute dot count values across all trials containing stimuli of the same coherence level. The average value of normalized DC is then computed across all trials related to the coherence level with the respective direction of correct alternative (right or left). Error bars indicate the standard error of the mean.

To quantify the spatiotemporal properties of the RDM stimuli (features), we propose a dot counting (DC) algorithm (Figure 8A) that approximates what dot motions the human brain may infer from the with random-dot motion stimuli. We computed the stimulus features, *dc*(*t*), for every trial in our experiment using the recorded, time-dependent dot positions (∼40 dots). The DC algorithm iterates through every dot for each time-step. Let us consider a dot with center coordinates of (*x_t_*,*y_t_*) at each time-step, *t*. If within a period of 50 ms after *t*, a new dot appears within a square area to the right of the current dot position (*x_t_*,*y_t_*) with the distance between the center of two dots below *dx*≅8 pixels horizontally, and *dy*≅*dx*/2 vertically, then the DC algorithm adds +1 to *dc*(*t*). Likewise, if the new dot appears within a square area to the left of the current dot then the DC algorithm adds −1 to *dc*(*t*) (see Figure 8A). The value of *dx* used in our implementation of DC algorithm was tuned to amount the of horizontal displacement for coherently moving dots between every two consecutive presentations of every set of dots that was used in the RDM stimulus generation algorithm.

Figure 8B demonstrates an example of DC stimulus feature for a zero coherence trial. A positive *dc*(*t*) value indicates net rightward motion whereas a negative value indicates net leftward motion within the stimulus aperture in the time-step, *t*. The DC algorithm captures the main features of the RDM stimuli: the direction and strength of coherent motion. Figure 8C shows the ratio of DC values to the total number of dots on the screen per time-step averaged across time steps and trials within a coherence level.

### Exact Input Model

To model the consistent responses with 0% coherence stimuli, we used an adapted version of the famous drift diffusion model (Ratcliff, 1978; Ratcliff and McKoon, 2008) that incorporates spatiotemporal stimulus features extracted using the DC algorithm. These dot counts entered the model via a scaling parameter that controlled the influence of the measured dot counts on the decision making process (scale equal to 0, no influence; large scale, large influence). The evidence accumulation of this adapted model is as below:


(1) $$D\,V_{t}-D\,V_{t-△\,t}=s\,c\,d\,c\,\left(t\right)\,△\,t+\sqrt{△\,t}\,s\,\varepsilon_{t}$$

where *DV*_*t*_ is the decision variable and *dc(t)* is the dot count value at the given time *t*, △*t* is the time-step length, *sc* stands for the scale parameter used to scale the dot counts, *s* is the diffusion rate, and ε_*t*_~*N*(0,1) is a standard normally distributed noise variable. Since the model scales the stimulus features in its evidence accumulation process, we called it the exact input model (EXaM).

Similar to the DDM, the EXaM describes the decision process in terms of a random walk with the difference that at each time step *t*, the increment of the decision variable has mean *sc dc(t) △t* whereas in the DDM it has time-independent mean *v*△*t*. In other words, the EXaM incorporates the time-dependent information encoded within the stimulus by replacing the trial-wise drift rate parameter *v* used by the DDM with the scaled dot count, *sc dc(t)*. Analogous to the variability of drift in the DDM, we further allowed the scale parameter *sc* to vary from trial to trial and drew trial-specific values for it from a Gaussian distribution: *sc* ∼ $N\,\left(\bar{s\,c},\sigma_{s\,c}\right)$. Note that the evidence accumulation process of the EXaM described by Eq. (1) is equivalent to the one of standard DDM if *dc(t)* is constant through time. Therefore, the EXaM can be adapted to a standard DDM in case stimulus information provided as an input to the model is constant.

**TABLE 3:** Free model parameters and their prior distributions.

Parameter	Prior	Prior parameters	
		Mean	Std
$\bar{\text{sc}}$	Zero	0	0.1
σ**_sc_**	Log-normal	−3	0.5
**B**	Log-normal	−3	1
${\bar{\text{z}}}_{0}$	Normal	0	0.5
**s_Z_**	Log-normal	−3.9	0.5
${\bar{\text{T}}}_{\mathrm{nd}}$	Log-normal	−2	1
**s_t_**	Log-normal	−2.5	1
π**_l_**	(cf. Figure 9A)	−1.65	1
π**_to_**	Uniform	0	1

*The scale (sc) has a different prior distribution for DDM and EXaM, See description of the EXaM for the meaning of parameters.*

**FIGURE 9:**
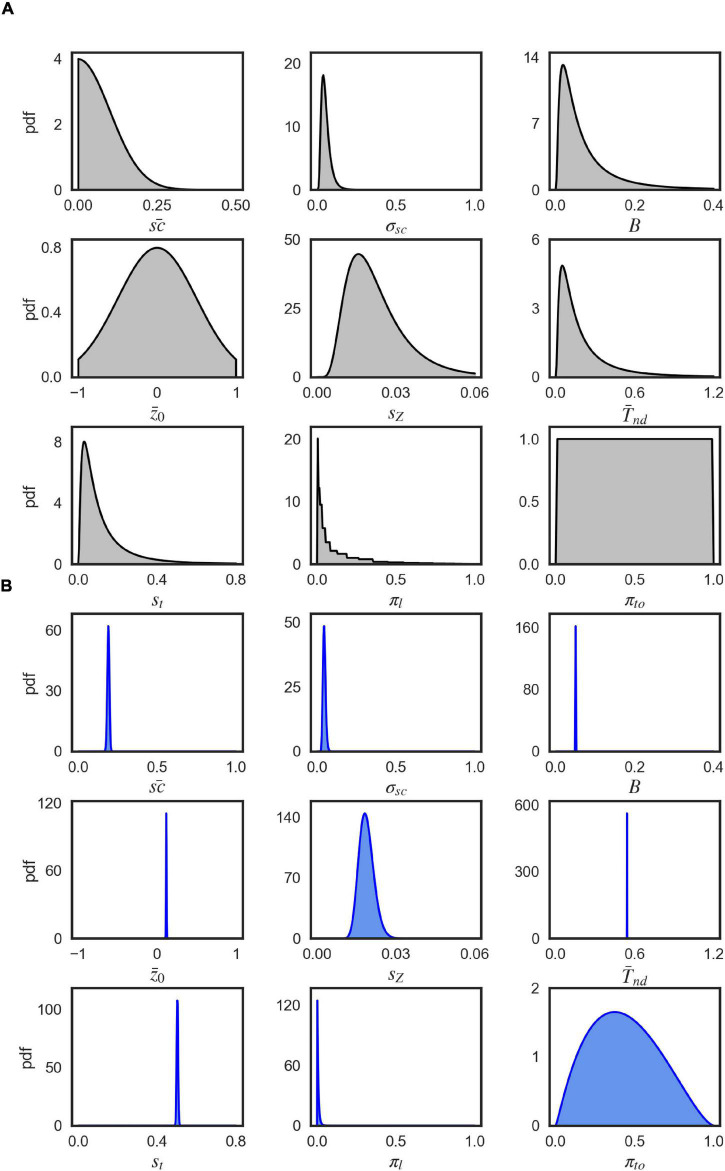
Visualization of parameter distributions. **(A)** Prior densities for each parameter in Table 3, **(B)** Example (marginal) posterior parameter densities of the EXM for participant 44, 0% coherence level.

The random walk and evidence accumulation process is concluded when the decision variable *DV*_*t*_ crosses one of the bounds at ±*B* which specifies the choice while the exact time-step of the crossing determines the reaction time. As in the DDM, the starting point, *DV*_0_ = *z*_0_, models the bias of a participant for one or the other alternative in each trial. It is drawn from a uniform distribution $z_{0}∼U\,\left({\bar{z}}_{0}-s_{z}/2,{\bar{z}}_{0}+s_{z}/2\right)$ in each trial where ${\bar{z}}_{0}$ is the mean bias and *s_z_* is the variability of bias. Furthermore, the EXaM includes an additive non-decision time modeling response delays due to basic sensory processing and motor production. This non-decision time, *T*_*nd*_, is drawn from a uniform distribution $T_{n\,d}∼U\,\left({\bar{T}}_{n\,d}-s_{t}/2,{\bar{T}}_{n\,d}+s_{t}/2\right)$ where ${\bar{T}}_{n\,d}$ is the non-decision time mean and *s_t_* is its variability. Finally, we have added two extra parameters to the conventional DDM formulation that model entirely random lapses. These parameters are called lapse probability π_*l*_ and time out lapse probability π_*to*_ (Park et al., 2016; see section “Materials and Methods” for more details) and give the probability with which a trial is modeled as a random lapse (random choice and RT drawn from uniform distributions) and the probability with which a lapse trial is timed out, respectively.

### Inference Over Models Given Behavioral Data

Note that, in the evidence accumulation process of the EXaM (Eq. 1), if the input term is constant across the trial (e.g., *dc*(*t*) = ±1 for every time-step *t*), then the term *sc dc(t)* is equal to the DDM’s drift rate which is constant within a trial. So, for our model comparison, we fitted the same model to the data and just manipulate the dot count input to implement the two models; for the EXaM, we used the actually measured dot counts, whereas for the DDM, we used constant *dc* = ±1 within every trial with sign determined by the true motion direction in that trial.

For inferring the model parameters and computing the marginal likelihood used in our model comparison, we used a Bayesian analysis method called EP-ABC (Barthelmé and Chopin, 2013). EP-ABC is a combination of Monte-Carlo inference (ABC) and variabitional Gaussian approximation applied on the posterior distributions of the parameters (EP). This method only relies on simulating from the model instead of using analytic definition of the model likelihood (see Barthelmé and Chopin, 2013 for more details).

We used a Python implementation of EP-ABC in our analysis, as available at: We ran EP-ABC with five independent repetitions for two models (DDM, and EXaM), for four coherence levels (0%, 10%, 25%, and 35% coherence levels), and each of the 44 participants. This leads to a total of 1,760 runs of EP-ABC. Our models had 9 free parameters (Table 3). The behavioral data (choice responses and RTs) were used to estimate the model parameters for each participant and coherence level. The method returned posterior distributions over parameter values and an estimate of the model evidence used for Bayesian model comparison.

As EP-ABC is a Bayesian method, it requires defining the prior distribution over model parameters. The applied implementation of EP-ABC only uses multivariate Gaussian distributions for its prior and posterior parameter distribution. Thus, we had to use parameter transformations to allow for non-Gaussian prior and posterior distributions. Similar to Park et al. (2016), we used the exponential and uniform parameter transformations. We used the exponential transformation to map a real value to a positive value and thus, transforming the Gaussian distribution into a log-normal distribution. Further, we used the uniform transformation to map a real value through the cumulative Gaussian density function and then scale and shift the values further. Thus, it transforms the standard normal distribution into a uniform distribution with a desired range. Also, when the transformation is applied to a different Gaussian distribution, it can also be used to introduce biases toward certain regions in the desired range. Additionally, to account for positive mean scale values, we have introduced a new transformation, the Zero transformation. The Zero transformation is a censored Gaussian distribution which maps a positive real value to itself and a negative real value to 0. Thus, it can concentrate a large proportion of probability mass at 0.

A priori, the parameters were uncorrelated so that we set the covariance between the prior parameters to 0. We defined the prior for each parameter by a univariate Gaussian distribution that was potentially combined with a transformation. Table 3 shows the particular setting for parameter priors that we used and Figure 9A shows the resulting univariate Gaussian distributions.

As a sampling-based method EP-ABC trades-off between the computational costs of the method and the quality of the approximate Bayesian inferences. The focus of our analysis was to select parameters that improve the quality of inference. The acceptance threshold was set to ε = 0.05. This means that when a response is sampled it is accepted only if it had the same choice response as the participant actually made in that trial and with the RT difference between sampled and actual responses not being more than 0.05 s. We set the minimum number of accepted samples to 2,000, the maximum number of samples per trial to 6,000,000, the alpha parameter to 0.5, and veps to 0.1. The EP-ABC procedure passed through the data twice. For meaning of these parameters, see Barthelmé and Chopin (2013) and the documentation of EP-ABC at http://github.com/sbitzer/pyEPABC.

EP-ABC returns the posterior distribution, as a multivariate Gaussian distribution, and the approximated model marginal likelihood (also called model evidence). To evaluate the parameter values in the original space used by the model, we sampled from the posterior distribution and used the respective transformation functions over the sampled values. The posterior parameter means that are reported in Tables 1, 2 and Supplementary Table 1 are the means of the transformed samples. Figure 9B demonstrates an example of the univariate slices of the posterior probability density.

### Bayesian Model Selection

We used the random-effect Bayesian model selection (RFX-BMS) procedure (Stephan et al., 2009; Daunizeau et al., 2014; Rigoux et al., 2014) to formally compare the models. The RFX-BMS procedure computes the protected exceedance probability and model frequency based on the marginal log-likelihood that was generated by EP-ABC. The RFX-BMS procedure is part of Variational Bayesian Analysis (VBM) toolbox^3^.

### Regression Analysis

We fitted a linear regression model to test whether dependent variables (such as in the Figures 6A,B and Supplementary Figure 3) could be explained by the independent variables. We used the MATLAB fitlm function of the statistics and machine learning toolbox to fit the linear model to data.

## Data Availability Statement

All the data and procedures used for behavioral analysis and computational modeling in this study is publicly available as Matlab (MathWorks) and Python code at https://github.com/pouyanrfard/EXAM-RDM.

## Ethics Statement

The studies involving human participants were reviewed and approved by the Ethics committee of the Dresden University of Technology. The participants provided their written informed consent to participate in this study.

## Author Contributions

PF, SB, SP, and SK designed the study and wrote the manuscript. PF acquired the data. PF, SB, and SK analyzed the data. All authors contributed to the article and approved the submitted version.

## Conflict of Interest

The authors declare that the research was conducted in the absence of any commercial or financial relationships that could be construed as a potential conflict of interest.

## Publisher’s Note

All claims expressed in this article are solely those of the authors and do not necessarily represent those of their affiliated organizations, or those of the publisher, the editors and the reviewers. Any product that may be evaluated in this article, or claim that may be made by its manufacturer, is not guaranteed or endorsed by the publisher.

## Footnotes

1
http://psychtoolboxorg/

2
http://scikit-learn.org

3
http://sites.google.com/site/jeandaunizeauswebsite/code/rfx-bms
