# Supplementary material for: Stochastic Motion Stimuli Influence Perceptual Choices in Human Participants
Source: Front Neurosci. 2022 Mar 2;15:749728. doi: 10.3389/fnins.2021.749728 (PMC8926215; doi:10.3389/fnins.2021.749728)
Supplement: Supplementary file 1 [file Data_Sheet_1.pdf]

Supplementary Material

Stochastic motion stimuli influence perceptual choices in human participants

Supplementary Figures and Tables:

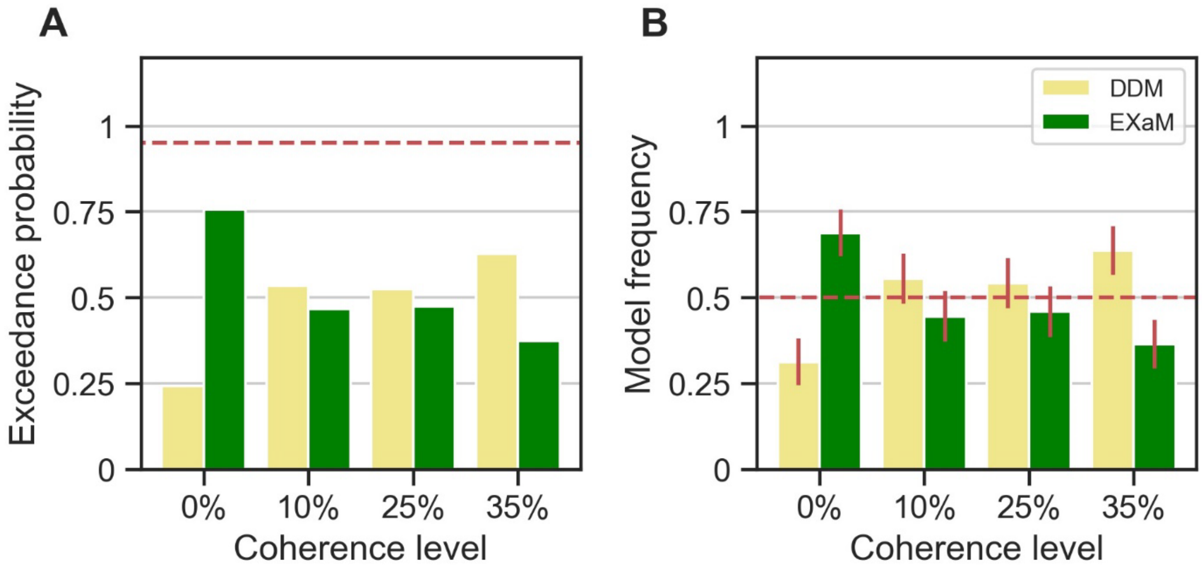

Figure S1. Results of random-effects Bayesian model comparison for all coherence levels across 44 participants, (A) Protected exceedance probability (probability that a model is the best model for all participants). The red dashed line indicates of very strong evidence for a model (0.95). **(B)** Model frequency for model comparison between DDM and EXaM. The model frequency is the probability that a randomly selected participant s behavior is best explained by a specific model, among the compared models. The red dashed line represents chance level and error bars indicate the standard deviation of the estimated model frequencies.

Table S1. Mean of the average posterior parameter distributions for the DDM and EXaM over the four coherence levels for 44 participants. Shown are the means over participants and the corresponding standard error in parentheses. The mean bias ($\bar{z}_0$) and variability of bias (s_Z) are both in terms of proportions of the bound (B) (ranging from -1 to 1). A mean bias of 1 indicates a complete bias towards the right choice and -1 a complete bias towards the left one. At the 0% coherence level, the parameter sc_1 indicates the scale parameter fitted to the trials containing Cluster 1 stimulus types, whereas the parameter sc_2 refers to the scale parameter fitted to trials containing Cluster 2 stimulus types. Asterisks indicate a significant difference between DDM and EXaM parameters for each condition (*p<0.05, **p<0.01, based on a paired t-test over 44 participants). See also description of the EXaM for the meaning of parameters.

	DDM				EXaM			
**Parameter**	0%	10%	25%	35%	0%	10%	25%	35%
$\overline{sc}_1$	0.02 (0.005)	0.16 (0.013)	0.31 (0.018)	0.39 (0.018)	0.01 (0.002)	0.06** (0.008)	0.09** (0.006)	0.10** (0.005)
$\overline{sc}_2$	0.01 (0.003)	-	-	-	0.037** (0.005)	-	-	-
σ_{sc}	0.05 (0.001)	0.06 (0.003)	0.06 (0.002)	0.06 (0.003)	0.053** (0.001)	0.06 (0.004)	0.05** (0.002)	0.05** (0.002)
B	0.05 (0.002)	0.07 (0.004)	0.07 (0.003)	0.07 (0.003)	0.05 (0.002)	0.07* (0.003)	0.09** (0.003)	0.09** (0.003)
$\bar{z}_0$	-0.13 (0.030)	-0.03 (0.025)	-0.04 (0.017)	-0.06 (0.015)	-0.025** (0.028)	-0.03 (0.023)	-0.05* (0.020)	-0.09** (0.018)
s_Z	0.02 (0.000)	0.02 (0.000)	0.02 (0.000)	0.02 (0.000)	0.02 (0.000)	0.02 (0.000)	0.02 (0.000)	0.02* (0.000)
$\bar{T}_{nd}$	0.81 (0.049)	0.59 (0.036)	0.42 (0.011)	0.42 (0.009)	0.79 (0.046)	0.56* (0.032)	0.44 (0.016)	0.44 (0.016)
s_t	0.63 (0.054)	0.44 (0.054)	0.09 (0.012)	0.08 (0.013)	0.59 (0.046)	0.40 (0.049)	0.16** (0.021)	0.17** (0.026)
π_l	0.07 (0.016)	0.04 (0.006)	0.03 (0.004)	0.03 (0.006)	0.06 (0.013)	0.02** (0.002)	0.02** (0.002)	0.01** (0.003)
π_{to}	0.34 (0.023)	0.30 (0.018)	0.35 (0.017)	0.35 (0.020)	0.34 (0.024)	0.37** (0.014)	0.40** (0.014)	0.41** (0.015)

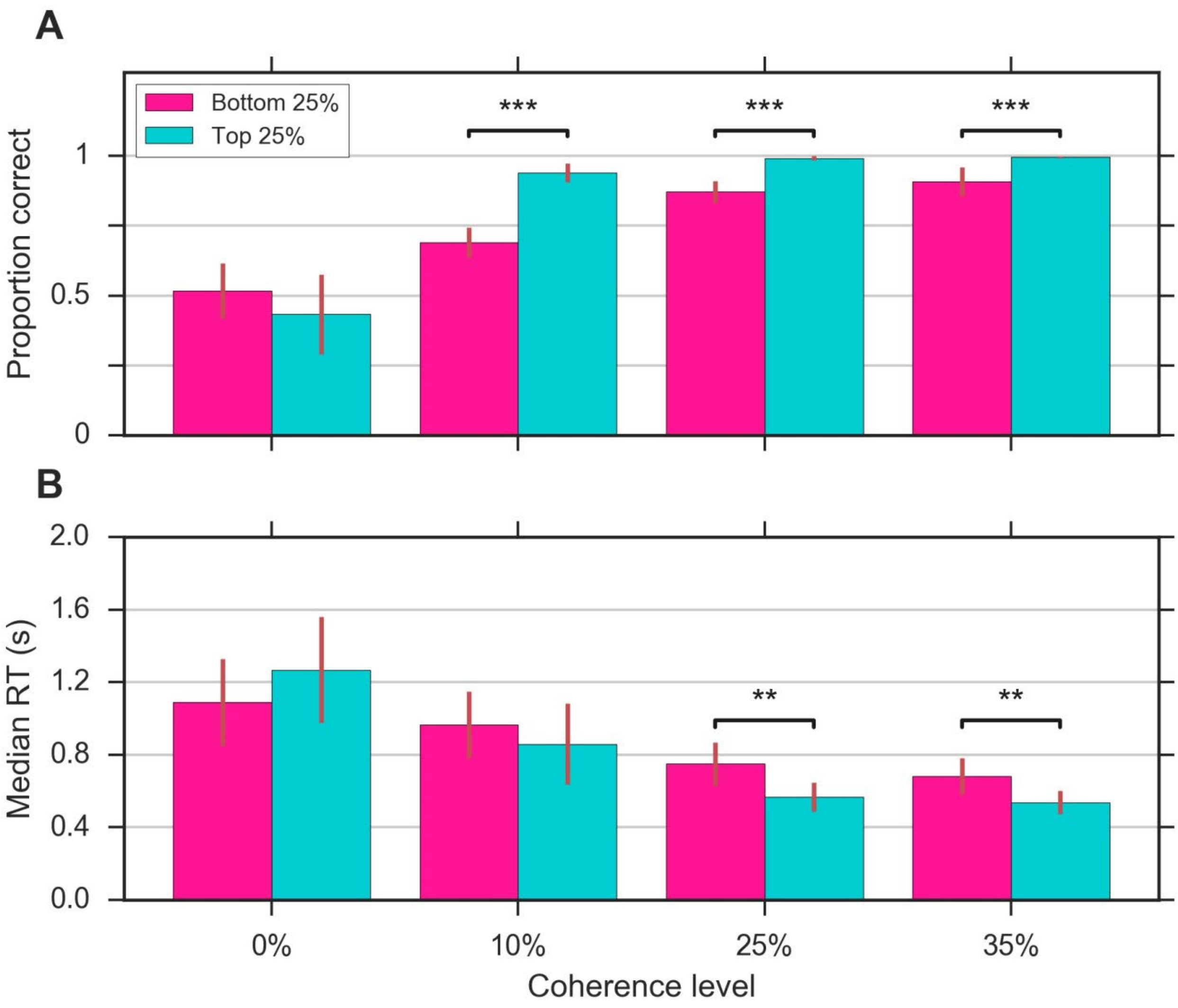
A
Bottom 25%
Top 25%
***
***
***
Proportion correct
1
0.5
0
B
2.0
1.6
1.2
0.8
0.4
0.0
Median RT (s)
**
**
0%
10%
25%
35%
Coherence level

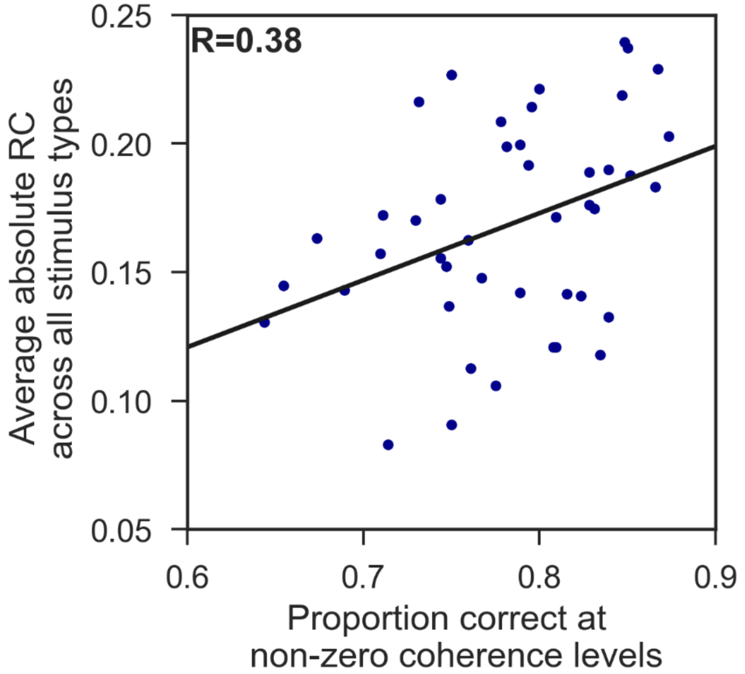

Figure S3. The relationship between non-zero % coherence proportion correct and response consistency. the average absolute response consistency across all stimulus types as a function of proportion correct at non-zero coherence levels (10%, 25%, 35%). The regression line shows a positive correlation between two variables with (linear regression Eq.2, $R = 0.38$, $p<0.05$).
